# Structural evolution and mechanism of strain glass transition in Ti_48.7_Ni_51.3_ shape memory alloy studied by anomalous small-angle X-ray scattering

**DOI:** 10.1038/s41598-020-66396-w

**Published:** 2020-06-10

**Authors:** Yung-Chien Huang, Cheng-Si Tsao, Shyi-Kaan Wu

**Affiliations:** 1 Dept. of Materials Science and Engineering, National Taiwan University, Taipei, 106 Taiwan; 2 Nuclear Fuel and Materials Division, Institute of Nuclear Energy Research, Taoyuan, 325 Taiwan; 3 Dept. of Mechanical Engineering, National Taiwan University, Taipei, 106 Taiwan

**Keywords:** Materials science, Nanoscale materials, Techniques and instrumentation

## Abstract

The *in-situ* anomalous small-angle X-ray scattering (ASAXS) technique was used to investigate the strain glass transition (SGT) in as-quenched Ti_48.7_Ni_51.3_ shape memory alloy during a thermal cycle of 30 °C to the SGT temperature *T*_g_ (−50 °C) and then to 30 °C again. The Ni atoms play a critical role as point defects in the SGT mechanism and are very difficult to characterize using conventional tools. ASAXS identified the distribution of Ni atoms in nanodomains, which have a disk-like core–shell configuration with a Ni-rich shell and a highly Ni-rich core. Moreover, the morphological evolution, growth and shrinkage of the highly Ni-rich core domains during the thermal cycle through *T*_g_ are demonstrated. The enhancement and reversible behavior of the local lamellar ordering arrangement of nanodomains during the SGT process at *T*_g_ are revealed. The structural evolution and local ordering arrangement of nanodomains can play a role in hindering martensitic transformation. The ASAXS results provide new knowledge about the SGT beyond that from current simulation works. However, this corresponding structure of the nanodomains was destroyed when the specimen was heated to 250 °C.

## Introduction

TiNi-based shape memory alloys (SMAs) exhibit excellent shape memory effects and superelasticity with a high damping capacity due to the well-known thermoelastic martensitic transformation^1^. The B19′ martensitic phase transformation from a dynamic strain-disordered B2 phase is attributed to long-range ordered (LRO) lattice strains. Remarkably, the regime of phase transformation of a ferroelastic system such as Ti_50-x_Ni_50+x_ SMAs changes from martensitic transformation to strain glass transition when x is larger than a critical concentration of 1.2 at.%^2–5^. The excess Ni atoms (called point defects or doping defects) play a critical role in inducing this crossover from normal martensitic transformation to strain glass transition. According to reported studies of TiNi SMAs^2,4,6–10^, strain glass demonstrates a strain liquid state (also called the unfrozen strain glass state), with most nano-strain domains dynamically flipping among possible strain orientations at temperatures above the glass transition temperature *T*_g_. With decreasing temperature, the nano-strain domains gradually grow, but they are eventually frozen into a statically strain-disordered state (called the strain glass state or the frozen strain liquid) below *T*_g_. In contrast to the LRO (martensitic or ferroelastic) transformation, the short-range ordered (SRO) strain glass transition is a conjugate transition of a ferroelastic system^2,3^. The strain glass experimentally exhibits a number of unusual properties^2–4,8,11,12^ as the signs of strain glass transition, such as (1) invariance of the average structure through *T*_g_, revealed by X-ray diffraction; (2) a frequency-dependent anomaly at *T*_g_, detected by dynamic mechanical measurement; (3) non-ergodicity in field-cooling and zero-field-cooling experiments; and (4) negative temperature coefficients of physical properties (electrical resistivity, thermal conductivity, etc.). Strain glass also demonstrates interesting properties in terms of the shape-memory effect and superelasticity^13^. An increasing number of strain glass systems (such as Ti–Ni–X and Ti–Pd–X, where X is an alloying element such as Fe, Cr, Mn, Co, V, or Cu) have been found, suggesting that strain glass is a general phenomenon in defect-containing ferroelastic systems^3,6–8,10,14,15^. Strain glass is a new horizon of ferroelastic systems and may offer opportunities for both fundamental research and new applications. However, the fundamental understanding the mechanism of such strain glass transition is still very limited.

Strain glass is characterized by random nano-strain domains. According to the existing phenomenological models and simulations of strain glass transition^3,6,9,16^, point defects (excessive dopant Ni atoms), or most probably defect pairs randomly distributed in the whole sample, form locally-ordered nano-strain domains and thus induce SRO strain at *T* < *T*_g_ to effectively inhibit the LRO or martensitic transformation^2,3^. These simulations suggest non-compositional transformation and fixed doped defects during strain glass transition. However, the existence of nano-strain domains in TiNi strain glasses was reported from observations by high resolution transmission electron microscopy (HRTEM)^3,4^. It is extremely difficult to use HRTEM to observe the real morphology of excess Ni-containing nanodomains because of their low electron density contrast. Therefore, the observed nanodomains may be mainly caused by the diffraction strain contrast from the lattice misfit. No HRTEM image of the real morphology of nano-strain domains has been published to date. On the other hand, the realistic morphology of well-defined nanodomains of as-quenched Ti_48.7_Ni_51.3_ SMA was recently confirmed due to the atom/electron density contrast (Δ*ρ*) between nanodomains and matrix through studies by small-angle X-ray scattering (SAXS) technique^17^ and neutron scattering (SANS) technique^18^. The advantage of SAXS or SANS technique is that the scattering intensity is not only contributed by the large number of domains but also is proportional to the product of (Δ*ρ*)^2^ and (domain size)^6^, leading to changes in intensity of orders of magnitude over the measured *Q* range^19,20^. The diameter (~50 nm) and thickness (~1.3 nm) of the disk-like morphology of well-defined nanodomains were determined in our previous SAXS study^17^. The roughly observed sizes of the randomly-distributed strain domains observed by TEM were 1–5 nm at room temperature (RT), 20–25 nm at *T* < *T*_g_ for the point-defect-induced domains^4^, and 20–40 nm at *T* < *T*_g_ for the precipitate-induced domains^21^ because the precipitate was thought to be composed of Ni atom defects. There still exists a gap in the relationship between the real well-defined nanodomains and nano-strain domains. In fact, the real spatial distribution at the large scale of the strain nanodomains at RT and the way the frozen nanodomains lead to the SRO strain in bulk glass when *T* decreases to *T*_g_ remain experimentally unknown due to the limitations of TEM and sampling.

For further in-depth study of the formation mechanism and the microstructural evolution of the real nanodomains through *T*_g_, it is necessary to solve some critical experimental challenges: (1) The actual distribution of the excess doped Ni defects in the well-defined nanodomains (formed during quenching) needs to be determined so that they can be accurately correlated with the nano-strain domains; (2) the variation of the Ni point defects and well-defined nanodomains during the strain glass transition needs to be examined. (3) Is the similar hindrance effect to LRO (martensitic transformation) or the formed SRO at *T*_g_ related to the variation of well-defined nanodomians? Direct experimental determinations to resolve these two challenges could yield actual solutions to the mechanisms, rather than the methods involved in examining nano-strain domains, the concept of which originated from simulation works. Compared with the current structural characterization tools, synchrotron *in-situ* anomalous small-angle X-ray scattering (ASAXS)^22–27^ with a tunable energy only sensitive to Ni atoms performed during a thermal cycle through *T*_g_ may be the most effective or even a unique approach for solving the above challenges. Basically, SAXS allows quantitative studies of the size, geometry and spatial orientations of a number of nanoparticles in the bulk sample, as has been demonstrated in various alloys^26,28–34^. In this study, the synchrotron *in-situ* ASAXS technique was performed during the thermal cycle (RT → *T*_g_ → RT→ annealing temperature) of as-quenched Ti_48.7_Ni_51.3_ specimens. The challenges mentioned above were successfully resolved, and the ASAXS results provide breakthroughs regarding the strain glass transition beyond the current simulation works.

## Results

### Temperature-dependent ASAXS measurement of the as-quenched Ti_48.7_Ni_51.3_ SMA

**Figure 1:**
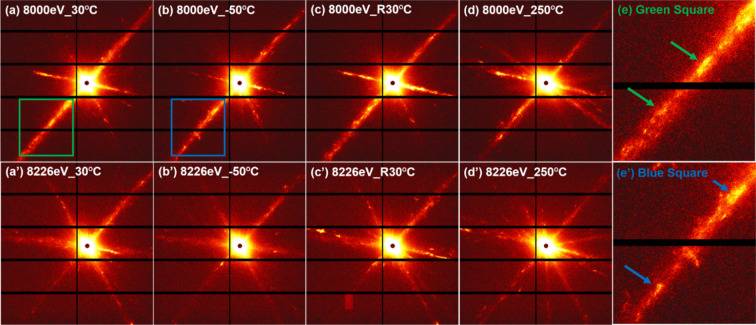
(**a**)(a’), (**b**)(b’), (**c**)(c’) and (**d**)(d’) 2D ASAXS patterns of the as-quenched Ti_48.7_Ni_51.3_ SMA measured using 8000 eV and 8226 eV at 30 °C, −50 °C, 30 °C (again) and 250 °C, respectively. (**e**)(e’) Magnified images of the green square and blue square in (**a**,**b**), respectively, using 8000 eV at 30 °C and −50 °C, to show bright spots along the streak by the arrows.

**Figure 2:**
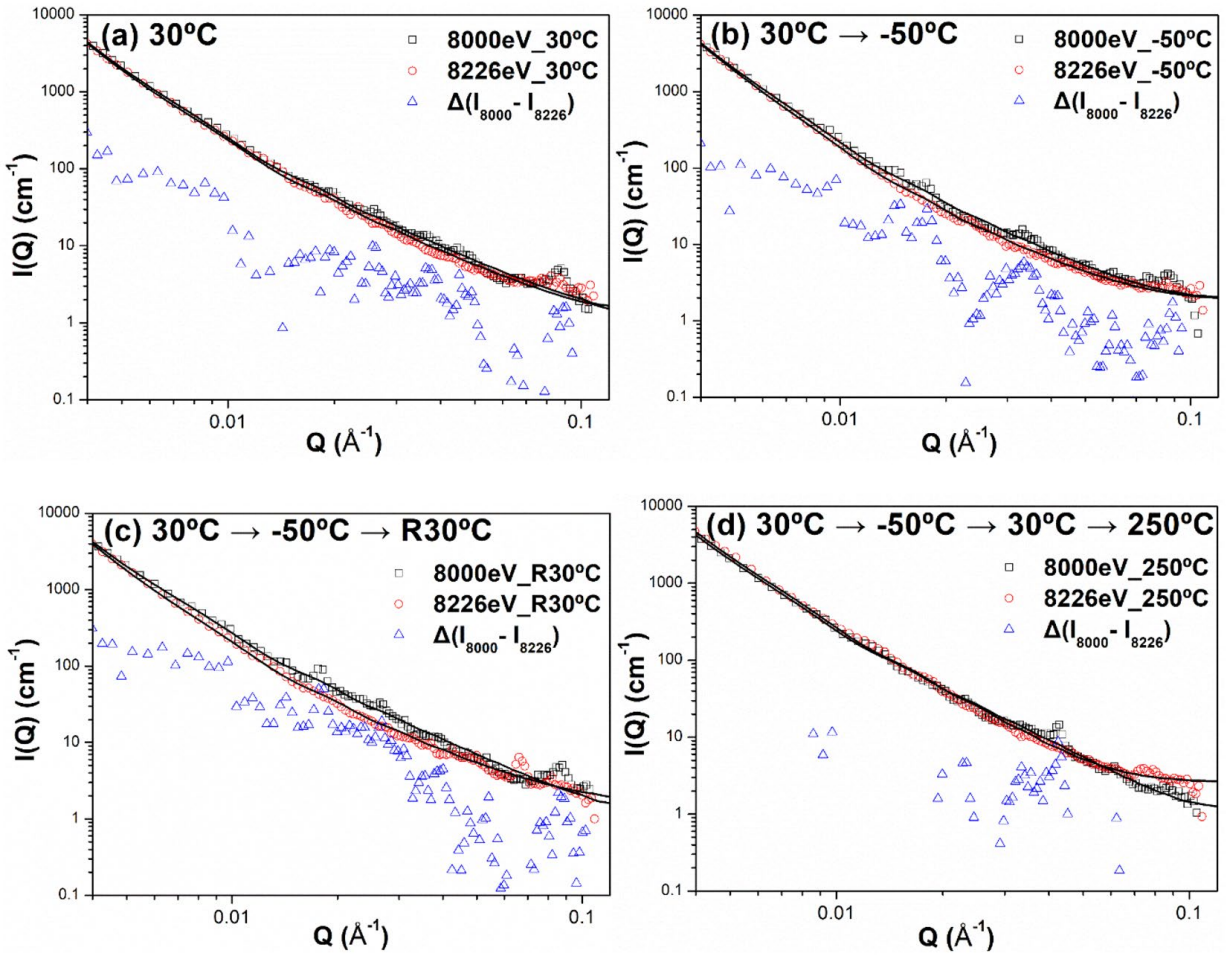
Evolution of 1D ASAXS profiles with the temperature measured at (**a**)30 °C, (**b**)−50 °C, (**c**)30 °C (again) and finally (d)250 °C. The model-determined ASAXS intensities by ***approach I*** are also shown by solid curves in diagrams for comparison. The standard deviations of each point associated with data are less than 2%.

Figure 1 (a)(a’), (b)(b’), (c)(c’) and (d)(d’) show the results of the temperature-dependent 2D ASAXS patterns of the as-quenched Ti_48.7_Ni_51.3_ SMA measured using 8000 eV and 8226 eV at 30 °C, −50 °C (at *T*_g_), 30 °C (end of cycle) and 250 °C (aging), respectively. The radial streaks in the 2D ASAXS patterns shown in Fig. 1 provide direct evidence of the plate-/disk-like morphology of the nanodomains^17,28,35–38^. The corresponding 1D ASAXS profiles are shown in Fig. 2. In that figure, the power-law scattering behavior (*I*(*Q*) ∝ *Q*^−2^) in the middle *Q* range (0.02–0.06 Å^−1^) of the ASAXS profiles also reveals direct evidence that the nanodomains are plate-/disk-like in shape^26^. The power-law scattering behavior of the exponent of −4 in the low *Q* range (0.003–0.009 Å^−1^) is contributed by the very large particles (surface scattering), which are beyond the scope of this study^26^. Also in Fig. 2, one can find that the 1D ASAXS profiles measured at different energies for the same temperature have similar profile shapes, except for some small peaks (i.e., structure peaks) only appearing in the ASAXS profiles measured at 8000 eV. Unlike the 1D ASAXS profiles without the structure peaks measured at 8226 eV (near the absorption edge of Ni), the structure peaks in the profiles measured at 8000 eV were apparently induced by Ni atoms in the nanodomains and thus provide evidence of Ni-rich nanodomains. These small structure peaks in the 8000 eV profiles could not be measured by the in-house SAXS instrument because its incident X-ray intensity was much lower than that of synchrotron X-ray radiation. On the other hand, Fig. 2 also indicates that two ASAXS profiles measured at two different energies substantially demonstrated a slight discrepancy between them, suggesting that the nanodomains were comprised of two components, (1) a Ni-rich component and (2) a highly Ni-rich component, in contrast to the Ti-Ni matrix. The present study further characterized the nanodomains to have a two-component structure by two approaches as follows.

## Structural characterization of core–shell disk domains in the as-quenched sample by ASAXS

### Model fitting of ASAXS intensities measured at two X-ray energies

**Figure 3:**
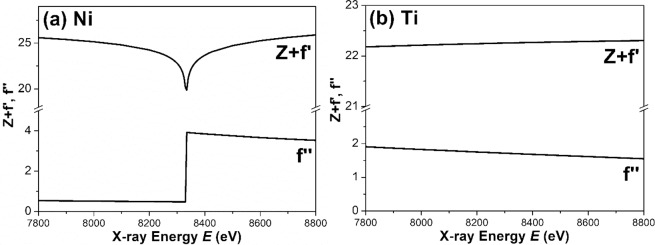
Coherent scattering, Z+ *f* ′, and absorption, *f* ″, parts of the scattering length of (**a**) Ni atom and (**b**) Ti atom in the vicinity of K absorption-edge of Ni atom.

The first proposed method of model fitting is called ***approach I*** here. The X-ray scattering amplitude (or length) of an atom, *f*(*E*), can be expressed by

1 $$f(E)=Z+f{\prime} (E)+if{\prime\prime} (E)$$

where *Z* is the atomic number and *f*(*E*) is a complex number. The anomalous dispersion correction terms *f*′ and *f* ″ are significant only near the absorption edge. The real part, *Z* + *f*′, is related to the coherent scattering. The imaginary part, *f* ″, is related to the absorption. The real and imaginary parts of the scattering length of Ni and Ti atoms as a function of incident X-ray energy *E* are shown in Fig. 3. Thereafter, the scattering length density of each phase, including the matrix and the Ni-rich and highly Ni-rich components in the nanodomains, can be obtained by dividing the average scattering length by the average atomic volume within the phase^27^:

2 $$\rho \,=\frac{{\sum }_{i}\,{f}_{i}{X}_{i}}{\Omega }$$

where *X*_*i*_ is the fraction of the atom *i* and Ω is the average atomic volume. The difference (or contrast) between the component phase and the matrix phase is given by^27^

3 $$\varDelta \rho =\frac{{\sum }_{i}\,{f}_{i}{C}_{i}^{p}-{\sum }_{i}\,{f}_{i}{C}_{i}^{m}}{\Omega }$$

where ${C}_{i}^{p}$ and ${C}_{i}^{m}$ are the concentrations of element *i* in the component *p* and the matrix *m*, respectively. Ω can be assumed to be identical in the component phase and matrix. Therefore, the ASAXS profile measured at 8226 eV can be modelled by

4 $$I(Q)={\rm{A}}\cdot {Q}^{-4}+\cdot {\rho }^{2}\cdot P(Q)\cdot S(Q)$$

where *A* is a constant describing the smooth surface of a large particle, Δ*ρ* denotes the scattering length density contrast between the nanodomains and the matrix, and *P*(*Q*) is the form factor of the nanodomains. *S*(*Q*) is the structure factor describing the interaction between the nanodomains and signifying a kind of ordered arrangement of nanodomains. *S(Q)* exhibits the form of several discrete small peaks whose amplitude of intensity is strongly affected by the incident X-ray energy or scattering contrast here. The form factor of the polydispersed disk-like nanodomains with a radius *R* and a Schulz distribution of thickness *t* can be given by^39–42^:

5 $$P(Q)=\frac{\eta }{{V}_{p}}\cdot {\int }_{0}^{\infty }{\int }_{0}^{\frac{\pi }{2}}{\left[2(\pi {R}^{2}t)f(t){j}_{0}\left(\frac{Qt}{2}cos\alpha \right)\frac{{j}_{1}(QRsin\alpha )}{(QRsin\alpha )}\right]}^{2}(sin\alpha )d\alpha dt$$

where $\eta$ and ${V}_{p}$ are the volume fraction and the particle volume, respectively, and *j*_0_ and *j*_1_ are the zero-order Bessel function and the first-order Bessel function, respectively. The integral over *α* averages the form factor over all possible orientations of the disks with respect to *Q*. *f(t)* is the normalized Schulz distribution of the thickness, in which the polydispersity of the thickness is related to *σ /t*_*avg*_, where *σ* is the variance of the Schulz distribution and *t*_*avg*_ is the mean thickness. Generally, *S(Q)* can be approximately 1.0 within the region outside the discrete peak. In the present study, the intensities of discrete small peaks of the structure factor *S(Q)* are much weaker than the form factor, so the model fitting of ASAXS profiles using Eq. (5) can ignore the discrete contribution of small structure peaks (let *S*(*Q*) = 1 for the full profile), unlike the continuous profile. The ASAXS profiles measured at different energies for all temperatures can be fitted well with Eq. (5), as shown by the solid curves in Fig. 2. The structure parameters determined by ***approach I*** are listed in Table 1. The determined polydispersity parameter is less than 0.05, showing the monodispersed system. There are certain uncertainties in model fitting.

**Table 1:** The structure parameters of the nanodomain are determined by the ***approach I***.

Incident energy	Parameter (nm)	Temperature			
		30 °C	−50 °C	R30 °C	250 °C
8000 eV	Radius	31.8 ± 0.1	34.0 ± 0.2	31.6 ± 0.1	36.1 ± 0.1
	Thickness	2.7 ± 0.3	4.3 ± 0.7	2.9 ± 0.7	4.4 ± 0.8
8226 eV	Radius	29.3 ± 0.1	32.8 ± 0.2	29.5 ± 0.1	35.0 ± 0.2
	Thickness	2.7 ± 0.4	4.1 ± 0.6	2.6 ± 0.6	4.4 ± 0.7

**Figure 4:**
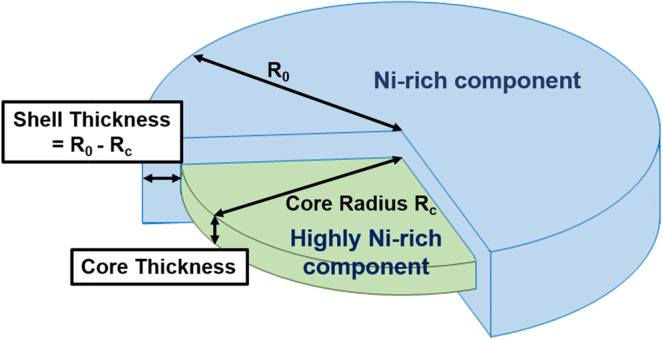
Core-shell model of the disk-like nanodomain.

According to Table 1, the determined thicknesses of the nanodomains for different energies are almost the same for each temperature. However, the values of the determined radii at 8000 eV for 30 °C, −50 °C and 30 °C again are larger than those determined at 8226 eV by a factor of ~2 nm. Therefore, based on this ASAXS analysis result, we can propose that the structure model of a nanodomain is a core–shell disk. The disk-like core–shell nanodomain is comprised of a highly Ni-rich disk as the core and a Ni-rich shell around it, as shown in Fig. 4. The shell thickness (the difference in radii of the inner disk and outer disk) for each temperature is ~2 nm. This thickness can be explained by the fact that the 8000 eV beam can “see” the morphology of the full core–shell disk, while the 8226 eV beam can only “see” the morphology of the core (highly Ni-rich domain). The difference between the determined structures is the shell (Ni-rich domain). Further explanation of this model fitting method is provided as follows. The form factor of the nanodomains, Eq. (5), can be alternatively expressed by the following equation.

6 $$I(Q)=\Delta {\rho }_{c}^{2}{P}_{c}(Q)+\Delta {\rho }_{s}^{2}{P}_{s}(Q)$$

where $\varDelta {\rho }_{c}^{2}$ and ${P}_{c}(Q)$ are the scattering contrast and the associated form factor of the core domain, respectively; $\varDelta {\rho }_{s}^{2}$ and ${P}_{s}(Q)$ are the scattering contrast and the associated form factor of the shell domain, respectively. As illustrated in Fig. 3, when the incident 8000 eV energy is switched to 8226 eV energy, *f*_*Ni*_ can be reduced by 4% and approach the value of *f*_*T*i_. Meanwhile, the *f*  ″_*Ni*_ and *f*_*Ti*_ (including *f*  ′_*Ti*_ and *f*  ″_*Ti*_) values remain unchanged. The Ni concentrations, *C*_*Ni*_ and *X*_*Ni*_, in the core domain are much higher than that in the shell domain. According to Eqs. (2) and (3), the scattering length density of the shell phase is much lower than that of the core phase. Therefore, $\varDelta {\rho }_{s}^{2}$ of Eq. (6) can be very small and be relatively neglected compared to the $\Delta {\rho }_{c}^{2}$ value, providing the explanation of why the incident 8226 eV energy only “sees” the core part.

## Model fitting of difference between ASAXS intensities measured at two X-ray energies

The present study also adopted another approach, ***approach II***, for cross-checking the above proposed ***approach I***. Because the different incident energies can change the atomic scattering length *f*, Eq. (6) can be expanded into the following equations:

7 $$I(Q,{E}_{1})=\Delta {\rho }_{H-Ni,1}^{2}{P}_{H-Ni}(Q)+\Delta {\rho }_{Ni,1}^{2}{P}_{Ni}(Q)$$

8 $$I(Q,{E}_{2})=\Delta {\rho }_{H-Ni,2}^{2}{P}_{H-Ni}(Q)+\Delta {\rho }_{Ni,2}^{2}{P}_{Ni}(Q)$$

where $I(Q,{E}_{1})$ and $I(Q,{E}_{2})$ are the ASAXS scattering profiles at *E*_1_ (8000 eV) and *E*_2_ (8226 eV), respectively. .. and ${P}_{i}(Q)$ are the scattering length density contrast and form factor of the domain *i* at the incident energy *j*, respectively. The subscripts *H-Ni* and *Ni* denote highly Ni-rich and Ni-rich, respectively. The atomic fraction of Ni in the Ni-rich shell can be regarded as only slightly higher than that of the matrix compared to the highly Ni-rich domains. The scattering contrasts of the Ni-rich domains for both energies may be approximately the same relative to those of highly Ni-rich domains. The scattering contrasts of highly Ni-rich domains for both energies are also significantly different. Therefore, the difference between the ASAXS profiles measured at *E*_1_ (8000 eV) and *E*_2_ (8226 eV) can be expressed as

9 $$I(Q,{E}_{1})-I(Q,{E}_{2})=(\Delta {\rho }_{H-Ni,1}^{2}-\Delta {\rho }_{H-Ni,2}^{2}){P}_{H-Ni}(Q)$$

**Figure 5:**
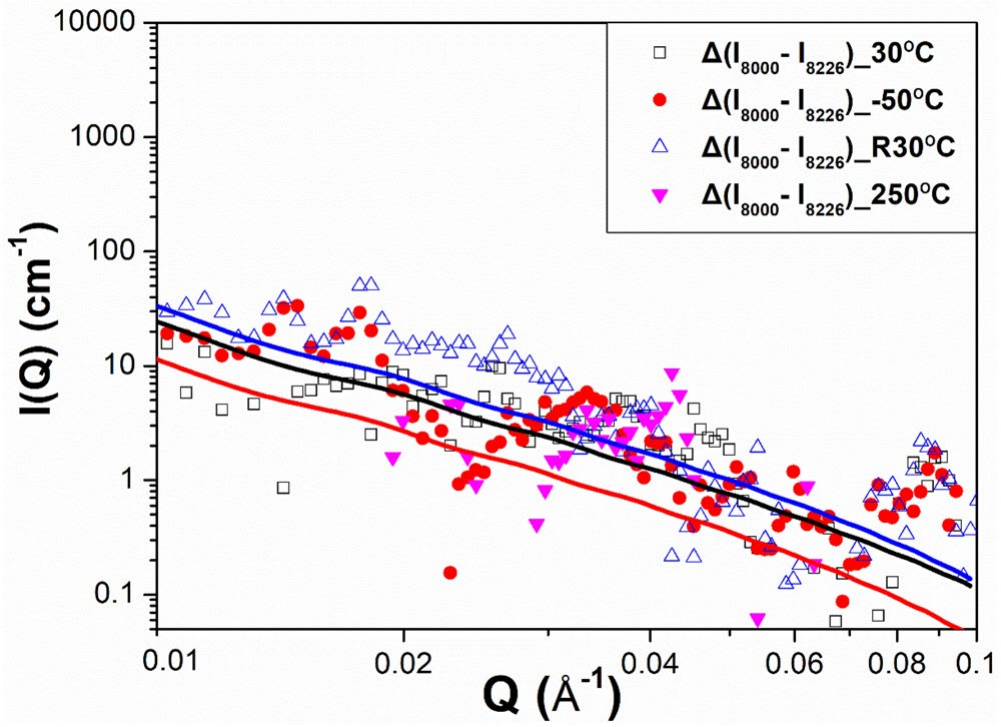
The difference between the ASAXS profiles measured at difference energies for the measurement at 30 °C, −50 °C, 30 °C (again) and finally 250 °C (heating). The model-determined ASAXS intensities by ***approach II*** (solid curves) are shown for comparison. The standard deviations of each point associated with data are less than 4%.

According to Eq. (9), the difference between the ASAXS profiles measured at *E*_1_ and *E*_2_ can be considered to be mainly contributed only by highly Ni-rich domains or by net scattering of the highly Ni-rich domains. This concept, exhibited in Eq. (9), has frequently been adopted in ASAXS analysis and is described in the literature^23–25^. *A****pproach II*** adopts the core–shell form factor model^26^ to directly fit the difference between the ASAXS profiles measured at 8000 eV and 8226 eV shown in Fig. 5, which is fitted well. The structure parameters determined by ***approach II*** are listed in Table 2.

**Table 2:** The structure parameters of the nanodomain are determined by the ***approach II***.

Δ(I_8000_-I_8226_)	Parameter (nm)	Temperature			
		30 °C	−50 °C	R30 °C	250 °C
Intensity difference between 8000 eV and 8226 eV	Core Radius	29.2 ± 0.2	32.0 ± 0.2	29.6 ± 0.2	—
	Core Thickness	2.6 ± 0.5	4.0 ± 0.8	3.0 ± 0.7	—
	Shell Thickness	2.0 ± 0.3	2.0 ± 0.7	1.7 ± 0.6	—

**Figure 6:**
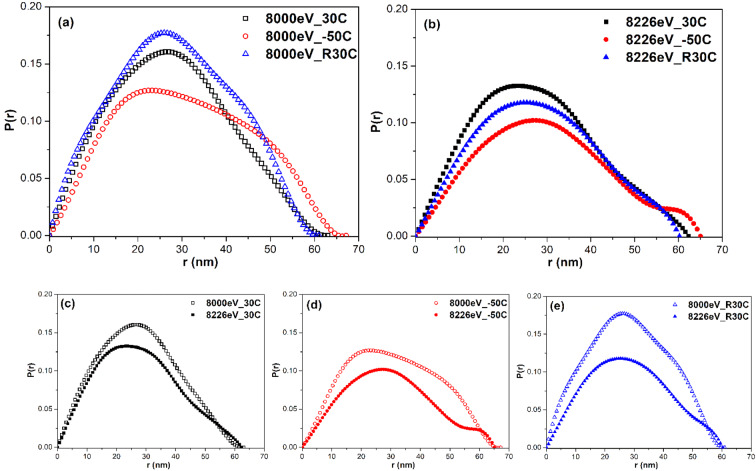
Distance distribution functions of the ASAXS profiles measured at (**a)** 8000 eV and (**b**) 8226 eV for each temperature. The comparison of *P*(*r*) functions measured at two energies for (**c**) 30 °C, (d) −50 °C, (**e**) 30 °C again.

Pair distance distribution function (PDDF) or DDF is a model-independent method to check the results obtained from the above model-fitting methods. The DDF, *P*(*r*), describing the real space of nanodomains can be directly calculated from the inverse Fourier transformation of the SAXS or ASAXS data according to $P(r)={r}^{2}{\int }^{}{Q}^{2}I(Q)[\,\sin (Qr)/Qr]dQ$. The *P*(*r*) functions corresponding to the SAXS profiles measured at 8000 eV for 30 °C, −50 °C and 30 °C again are shown in Fig. 6(a). The *P*(*r*) functions corresponding to the ASAXS profiles measured at 8226 eV for 30 °C, −50 °C and 30 °C again are shown in Fig. 6(b). The comparison of *P*(*r*) functions measured at two energies for 30 °C, −50 °C and 30 °C again are shown in Fig. 6(c–e), respectively. The typical curve of *P*(*r*) for a disk-like particle shows two characteristics: (1) The maximum *r* position corresponds to the diameter of this particle, and (2) the peak position of *P*(*r*) is close to but less than the radius of this particle^43^. According to Fig. 6(a,b), for either 8000 eV or 8226 eV, the maximum *r* positions (domain diameter) of *P*(*r*) for 30 °C, −50 °C and 30 °C again are located at ~ 60 nm, 65 nm and ~60 nm, respectively, being roughly consistent with the model-fitting result of ***approach I*** in Table 1. The variation trend of domain size with temperature is the same for all used methods. The area-averaged peak (roughly close to the domain radius) of *P*(*r*) for 30 °C, −50 °C and 30 °C again are located at ~ 28 nm, 30 nm and ~28 nm, respectively, also showing the same relative trend. According to Fig. 6(c–e), the area-weighted peak position of 8000 eV is higher than that of 8226 eV for each temperature, showing the evidence of core-shell structure.

### Structural transition and ordering behavior revealed by the *in-situ* ASAXS structure factor under the thermal cycle

**Figure 7:**
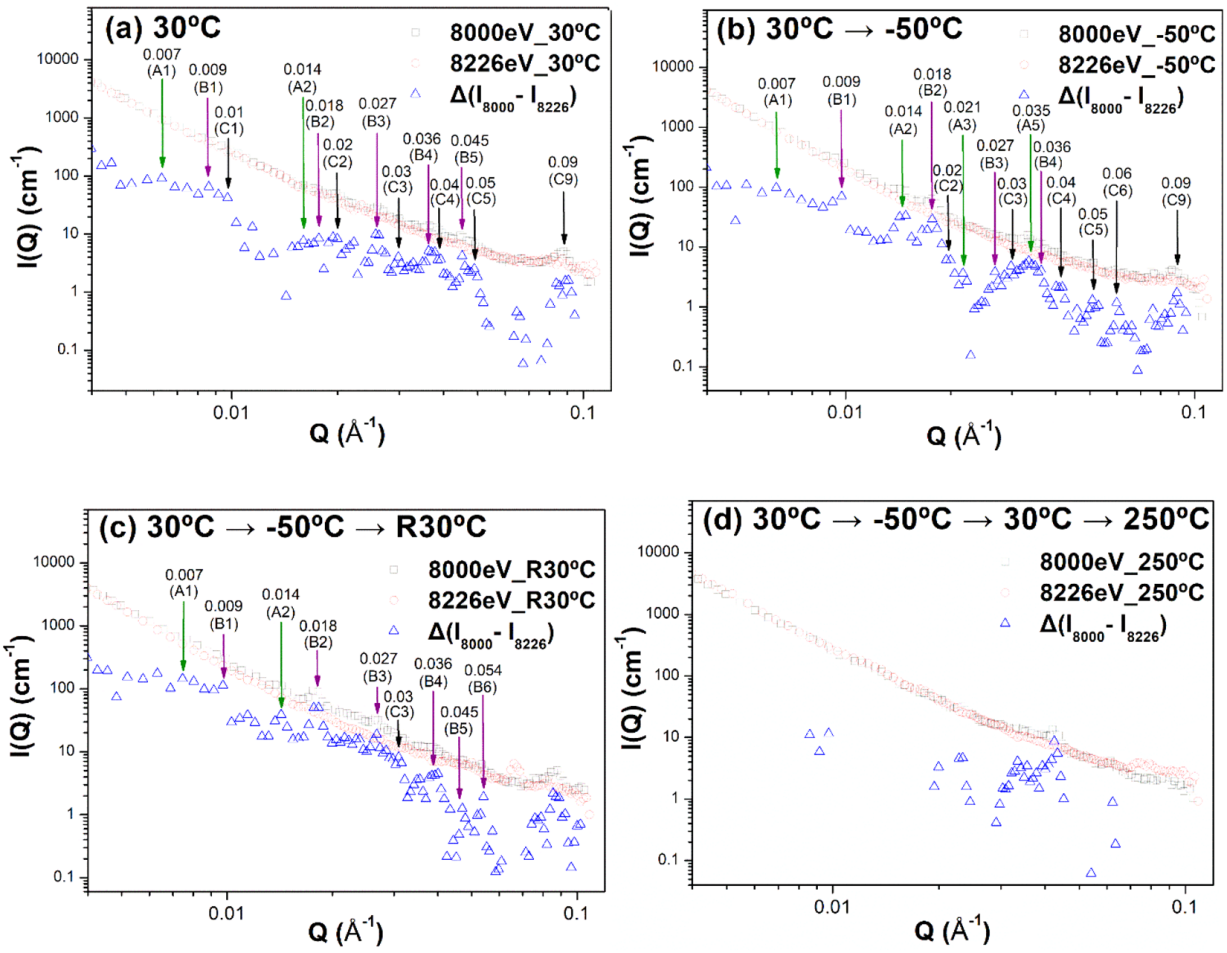
ASAXS profiles at 8000 and 8226 eV in comparison with the difference between ASAXA profiles at (**a**) initial 30 °C, (**b**) −50 °C, (**c**) 30 °C again and (**d**) 250 °C in order during the thermal cycle. Each structure peak is marked (For example, A2 represents the second-order peak of group A).

The morphological characterization and evolution of the core–shell nanodomains during the thermal cycle are revealed by the form factor analysis of ASAXS profiles in the previous sections. This morphological evolution should be closely related to the movement of Ni atoms governed by temperature change. This behavior of Ni atomic movement into/out of the nanodomain core can be independently evidenced by the change in height of the structure peaks (also called the structure factor) within the ASAXS profiles shown in Fig. 2. Basically, the structure factor results from the interaction between domains or the spatial arrangement of domains^26,42^. These relatively weak structure peaks are marked in detail by the arrows shown in Fig. 7 and do not appear in the SAXS profiles measured by the in-house X-ray instrument^17^. This difference can be attributed to the high resolution and high intensity of synchrotron X-ray radiation. The small structure peaks caused by the spatial arrangement of the concentrated Ni-rich domains are easily self-attenuated in the bulk sample, leading to difficulty in detection. These structure peaks are also detected in the form of bright spots in the radial streaks of the 2D ASAXS patterns, as marked in Fig. 1(e) (e’). The relative positions of these structure peaks of the as-quenched TiNi sample reveal the relationship of integer times. The *n*^th^ order peak follows the first order peak with a rough position ratio of 1: 2: 3: 4: … : *n*. For example, from Fig. 7, the position of the first order peak for group A is 0.007 Å^−1^, that of the second order peak is 0.014 Å^−1^, and so on. This relationship agrees with the prediction of the structure factor of concentrated lamellar stacks with periodic distance^44,45^. According to the general concept of the ordered lamellar structure, the average distance between lamellae (or plates) *D* can be approximated by *D* = 2π/*Q*_1_. Here, the *Q*_1_ value is the peak position of the first-order peak. Therefore, it can be proposed that the several plate-like nanodomains construct an ordered arrangement with equal spacing (like the structure of lamellar stacks).

## Discussion

The ASAXS profiles shown in Fig. 2 could be fitted by two possible form-factor models. One is the disk form factor to depict the whole disk of the nanodomain, comprising the shell and core components, at 8000 eV and only the core component at 8226 eV, followed by constructing the real morphology with a Ni-rich shell and highly Ni-rich core, denoted by ***approach I***. The other is the form factor of the core–shell disk model^26,42,46^ with two scattering contrast values directly fitting the profile resulting from the difference between the ASAXS profiles at 8000 eV and at 8226 eV, denoted by ***approach II***. Both single disk and core–shell form factors can fit well the continuous scattering profiles under ignorance of *S*(*Q*), as shown in Figs. 2 and 5. The corresponding structure parameters determined by ***approach I*** and ***approach II***, shown in Tables 1 and 2, respectively, are close to each other. The core radius *R*_*c*_ and thickness *t*_*c*_ of core-disk nanodomains determined by ***approach II*** grew from *R*_*c*_ = 29.2 nm and *t*_*c*_ = 2.6 nm at 30 °C to be 32.0 nm and 4.0 nm, respectively, when the irradiated sample was cooled down to −50 °C (at *T*_g_). Moreover, the *R*_*c*_ and *t*_*c*_ of core-disk nanodomains shrank to 29.6 nm and 3.0 nm, respectively, when the irradiated sample was subsequently heated to 30 °C. The shell thickness under the thermal cycle (30 °C → −50 °C → 30 °C end) remained at about 2 nm. The fitted radius (~26 nm) of nanodomains in our previous SAXS result^17^ was less than that (~ 30 nm) of this synchrotron ASAXS analysis. The discrepancy is attributed to the *Q*_min_ of the *Q* range in this ASAXS study having a much lower value and accounts for the greater power-law scattering intensity than that of the SAXS data of the previous SAXS measurement. If the fitted ASAXS data had been limited to a short *Q* range and had no power-law model fitting, we would have had the same fitting result as the previous result. In this study, we found that the morphological change (growth) of the highly Ni-rich domain (core part) occurs when the sample is cooled from 30 °C towards *T*_*g*_. This morphological change is reversible in the sample heated back to 30 °C. Notably, the structural evolution and then the reversible behavior of the highly Ni-rich disk nanodomains exhibited in as-quenched Ti_48.7_Ni_51.3_ SMA during this thermal cycle of 30 °C → −50 °C (~ *T*_*g*_) → 30 °C (end) are evidenced in this ASAXS study for the first time. This behavior has never been mentioned in the existing theory, in which the strain-glass transition is proposed to arise from the growth of the strain field in the matrix based on the assumption of a uniformly unchanged distribution of Ni point defects^2,4,8^.

As mentioned above, the model constructed from the combination of the ASAXS profiles measured at 8000 eV and 8226 eV, and that constructed directly from the difference in ASAXS profiles measured with different energies, can both substantially reflect the characteristics of the highly Ni-rich part. According to Fig. 7(a), the structure peaks revealing the lamellar stacks and the spatially ordered arrangement with equal spacing in the as-quenched sample could be divided into three groups, denoted by A, B, and C. The spots in the streaks of the 2D ASAXS patterns show that they originate from differently-oriented grains. The *Q*_1_ values of groups A, B and C are 0.007 Å^−1^, 0.009 Å^−1^ and 0.01 Å^−1^, respectively. The corresponding spacing *D* for groups A, B and C (*D* = 2π/*Q*_1_) can be determined to be 897 Å, 698 Å and 628 Å, respectively. As shown in Fig. 7, each group has several structure peaks, e.g., A1, A2, …, demonstrating the characteristic of the highly ordered packing of the nanodomains. The observed first-order peaks (in logarithm scale) are not sharp because they are located in the high background contributed by the form factor. The spatially ordered lamellar arrangement of nanodomains may be formed during the quenching process and could be closely related to the critical Ni composition. More interestingly, when the temperature is cooled to *T*_*g*_, as shown in Fig. 7(b), the intensities of the first- and second-order structure peaks of each group largely increase and become sharp in shape, which can be attributed in theory to the increase in the scattering length density contrast Δ*ρ* of the core domain as well as the number of the ordered lamellae. The increase in Δ*ρ* of the core domain may be caused by the short-range movement of Ni atoms into the core domain, which is consistent with the morphological growth of the core domain at *T*_*g*_ (determined by the form factor in the previous sections). This local action may enhance the number of the “seen” (by contrast) nanodomains in the ordering arrangement based on the assumption that the total number of nanodomains during the thermal cycle is not changed. This kind of local Ni movement and ordering arrangement of nanodomains occurs quickly in the *in-situ* ASAXS measurement during the thermal cycle. Therefore, the kinetics of Ni movement and the core-domain growth at *T*_*g*_ are fast. It can be speculated that the driving force of structural transition is not classical thermal diffusion. The driving force may be the free energy and could be similar to that of the formation of the spinodal decomposition, which has the characteristic of fast movement from a low concentration to a high concentration^47,48^. The mechanistic origin and the driving force are still not known and need to be further investigated.

When the temperature returns from −50 °C (~*T*_*g*_) to 30 °C, except for in group B, the structure peaks of the other groups become weak in intensity and less in number (Fig. 7(c)), showing the phenomenon that Ni atoms move out of the nanodomains and the number of the “seen” (by contrast) nanodomains in the ordered arrangement decreases. The structural evolution of the strain glass transition appears be possibly reversible with heating to 30 °C, but the material cannot return to its original state. The thermal effect on nanodomains at a higher temperature of 250 °C causes almost all of the structure factor peaks to disappear (Fig. 7(d)), signifying that a large number of Ni atoms move out from the nanodomains and thus cause the loss of ordering arrangement or lamellar stacks. The large amount of Ni atoms thermally diffusing in the matrix could serve as the source of the precipitation at high temperature, according to the classical mechanism of nucleation and growth. The form factor analysis in the previous sections consistently shows that the nanodomain rapidly grows in radius and thickness, as shown in Table 1. The precipitation of Ti_48.7_Ni_51.3_ SMA at the annealing temperature of 250 °C was quantitatively reported in our previous study^17^. From this study, one can find that, for as-quenched Ti_48.7_Ni_51.3_ SMA, the structural evolution mechanism (ordering array of nanodomains) between 30 °C and −50 °C (*T*_*g*_) is entirely different from the precipitation mechanism exhibited at 250 °C.

## Conclusions

Synchrotron *in-situ* ASAXS study provides new mechanistic and structural knowledge about the strain glass transition exhibited in as-quenched Ti_48.7_Ni_51.3_ SMA. Past simulations and phenomenological models proposed that the induced SRO strain in the glass state was due to the statically-disordered strain domains in the bulk material, based on the assumption that randomly-distributed Ni point defects were fixed in position during strain glass transition. Our ASAXS study provides evidence that Ni point defects concentrate in well-defined nanodomains, with a disk-like core–shell structure consisting of a highly Ni-rich core and a Ni-rich shell in as-quenched Ti_48.7_Ni_51.3_ SMA. In addition, at 30 °C, these disk-like nanodomains are locally ordered with a spatially lamellar arrangement. When the temperature decreases to *T*_*g*_, some Ni point defects in the matrix move a short distance into the nanodomains to enlarge the size of the highly Ni-rich core and enhance the ordered structure of the local lamellar arrangement with increased numbers. When the temperature rises back to 30 °C, the morphological evolution of the highly Ni-rich core domains with temperature reveals reversible growth and shrinkage. At 250 °C, the nanodomains grow but lose the corresponding lamellar structure, and precipitation occurs. For as-quenched Ti_48.7_Ni_51.3_ SMA, the phase separation mechanism in the range of 30 °C to −50 °C (*T*_*g*_) is entirely different from the precipitation mechanism exhibited at 250 °C. The ASAXS patterns measured with the near Ni adsorption edge energy demonstrate the structure peaks, signifying the local order arrangement between nanodomains and the associated variation with *T*_g_ during thermal cycle. This new finding can be regarded to be an experimental breakthrough compared to the current SAXS, SANS and microscopic tools. Therefore, the similar hindrance effect on LRO or the formation of SRO at *T*_g_ proposed by simulation works may be closely related to the enhancement of the local lamellar or ordering array as well as the morphological variation of the nanodomains.

## Experimental procedures

A Ti_48.7_Ni_51.3_ ingot was prepared in a vacuum arc remelter. The raw materials, titanium (99.99 wt.%) and nickel (99.99 wt.%), were melted six times in high-purity argon atmosphere. The ingot was then hot rolled at 900 °C into a plate of ~2 mm thickness. The plate was subsequently solid-solution treated at 900 °C for 1 h, followed by water-quenching. The grain size in as-quenched Ti_48.7_Ni_51.3_ specimens is measured by ASTM E112–88 standard to be 80 ± 15 μm. The specimens for the following ASAXS measurements were cut to a size of 10 × 10 × 0.5 mm^3^ and ground to a thickness of ~40 μm so that two or more grains would be contained in the thickness direction. The detailed information on the sample preparations and the experiment conditions can be seen in our previous study^17^.

*In-situ* ASAXS measurements were conducted at the BL23A beamline, National Synchrotron Radiation Research Center, Taiwan, with a high-flux collimated X-ray beam of ~500 μm in diameter. The temperature-dependent ASAXS measurement was performed with two incident energies of 8000 eV and 8226 eV, which are the far and near absorption K-edge energies of Ni, respectively, for each temperature. The measurements were carried out to investigate the evolution of the Ni-rich nanodomains with temperature and the spatial distribution of Ni atoms in the corresponding Ni-rich nanodomains. The ASAXS measurement of the as-quenched Ti_48.7_Ni_51.3_ SMA was performed *in-situ* first at 30 °C, then at the cooled temperature of −50 °C, and finally at the heated temperature of 30 °C again to complete one cyclic test. According to the results of the physical properties and the DMA tests in our previous study^17^, a sample at −50 °C demonstrates the signs of strain glass transition. Moreover, a subsequent ASAXS measurement was performed at 250 °C after the specimen was aged at 250 °C for 20 min. Each 2D ASAXS pattern was collected for a period of 20 min. According to standard procedures of data reduction, calibration, and background correction, each pattern was azimuthally averaged into a 1D ASAXS profile as a function of the scattering vector *Q* = 4πsin(*θ*/2)/*λ*, in which *θ* is the scattering angle and *λ* is the X-ray wavelength.
